# Ischemic Stroke following COVID-19 in a Patient without Comorbidities

**DOI:** 10.1155/2021/8178529

**Published:** 2021-11-01

**Authors:** Rizaldy Taslim Pinzon, Maria Devita Kumalasari, Hana Kristina

**Affiliations:** ^1^Duta Wacana Christian University School of Medicine, Yogyakarta, Indonesia; ^2^Bethesda Hospital, Yogyakarta, Indonesia

## Abstract

**Background:**

Stroke is a rare complication of COVID-19. Post-COVID-19 stroke occurs mainly in older patients who have preexisting vascular risk factors. Most strokes are possibly related to hypercoagulability associated with COVID-19 where elevated D-dimer levels were the most common finding. In this case, post-COVID-19 ischemic stroke occurred in a relatively young patient without preexisting cerebrovascular risk factors which were rarely reported before. *Case Presentation*. A 40-year-old male presented lack of concentration, sluggish mind, and forgetfulness. The patient has a positive COVID-19 history 5 weeks ago. The noncontrast MSCT scan confirmed multifocal lacunar cerebral infarction on the left lateral ventricle. Laboratory tests showed an increase in D-dimer of 1.22 g/ml.

**Conclusion:**

In COVID-19 patients without comorbidities, ischemic stroke should be considered.

## 1. Introduction

COVID-19 caused by the COVID-19 virus is associated with multisystem dysfunction covering any neurologic involvement. The neurological involvement in the form of stroke as a clinical manifestation was reported in 6 patients (2.8%) with COVID-19 according to a study in Asia with 214 patients [1].

Stroke is a rare complication of COVID-19 with an estimated prevalence of 3% [2]. The incidence of stroke is reported to be higher among patients with COVID-19 than in the non-COVID-19 population. A preliminary case-control study showed that COVID-19 is an independent risk factor for acute ischemic stroke [3]. A stroke occurs mainly in patients with severe COVID-19 and who have preexisting vascular risk factors [4].

COVID-19 patients have significantly higher rates of cryptogenic stroke (stroke of undetermined etiology on TOAST criteria) than non-COVID-19 patients [5]. Most cryptogenic strokes are possibly related to acquired hypercoagulability obtained during COVID-19 [6]. In coagulopathy conditions associated with COVID-19, elevated D-dimer levels were the most common finding [7]. The long-term effect of COVID-19 on the elevated D-dimer is estimated to reach 20% [2].

## 2. Case Presentation

A 40-year-old male came to the neurology department with complaints of lack of concentration, sluggish mind, and forgetfulness. The patient has a positive COVID-19 history and is currently tested negative. Complaints have been felt since 6 days after the onset of COVID-19 symptoms and continued 5 weeks since the onset of acute COVID-19. The patient started experiencing symptoms of acute COVID-19 without respiratory symptoms. Initial complaints were fever, anosmia, and fatigue. Fever varied between 38.5 and 40.5°C. During the acute COVID-19 phase, the patient was isolated at the Wisma Atlet, Jakarta. The patient's O_2_ saturation had dropped to 88%, and then, he received oxygen therapy with a nasal cannula. He experienced a memory deficit in which he did not recognize his family members. The results of the MoCA-Ina examination showed a score of 30 meaning that there is no cognitive impairment.

**Figure 1:**
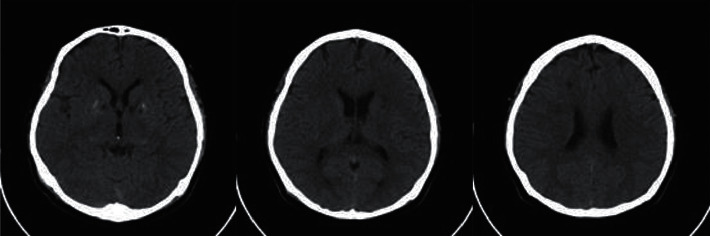
Multiple lacunar hypodense lesions on the CT scan.

The patient did not show weakness in the legs or arms. On neurological examination, including motor, sensory, and cranial nerve functions, no neurologic deficits were found as commonly found in stroke. The patient had no history of hypertension, diabetes, heart problems, stroke, TIA, thromboembolism, or other vascular diseases. Medical, family, and psychosocial history were normal. However, the noncontrast MSCT scan confirmed the presence of hypodense lesions on the right lateral ventricles and calcifications on the pineal body and lentiform nucleus. It can be concluded that CVA was obtained with multifocal lacunar cerebral infarction, especially on the left lateral ventricle (Figure 1).

Laboratory tests were performed 5 weeks after the onset of acute COVID-19 and showed an increase in D-dimer of 1.22 g/ml. The other laboratory parameters for stroke panel were normal (cholesterol, LDL, triglyceride, blood glucose level, and hemoconcentration). Pharmacological therapy was provided in the form of oral anticoagulants rivaroxaban (10 mg), citicoline (500 mg), vitamin D3 (1000 IU), and vitamin B complex. The follow-up showed significant improvement, and the patient became fully independent with modified Rankin Scale measurement.

## 3. Discussion

This study reports a case of ischemic stroke with multiple lacunar infarcts in patients with a history of COVID-19. COVID-19 was confirmed by the Polymerase Chain Reaction (PCR) test, and the diagnosis of ischemic stroke was confirmed by an MSCT scan of the head.

Besides the presentation of ischemic stroke, other complaints are prolonged fatigue, difficulty in concentrating, and memory problems. In self-isolated patients, the most common symptoms of physical exhaustion were fatigue (35%), increased need for rest (30%), and lack of energy (29%), while the most common symptoms of mental exhaustion were difficulty in finding words (23%), difficulty in concentrating (19%), and memory problems (18%). More than half of COVID-19 patients with mild to moderate illness experience those symptoms 6 months after the infection [8].

The onset of neurological symptoms ranges from 3–14 days from the onset of COVID-19 symptoms. It is in line with the Global COVID-19 registry (the average delay between the onset of COVID-19 symptoms and the onset of stroke is 7 days) [9]. In a study in New York, the median age of stroke patients was 62.5 years and the median time between the onset of the first COVID-19 symptoms and stroke was 10 days [6]. Referring to the Global COVID-19 registry, the imaging characteristics of stroke in COVID-19 involve 22.7% large vessels and 7.6% lacunar [9].

In this case, post- COVID-19 ischemic stroke occurred in a relatively young patient (40 years) without preexisting cerebrovascular risk factors which were rarely reported before. Considering the age of the patient and no accompanying cerebrovascular risk factors can help show that COVID-19 is an independent risk factor for acute ischemic stroke [10].

Early-onset cerebrovascular disease is more common in COVID-19 patients with underlying cerebrovascular risk factors including older age (>65 years) and very few reported cases of stroke in younger patients (<50 years) [11]. Based on the TOAST guidelines, specific vascular risk factors for ischemic stroke are diabetes mellitus (DM), hyperlipidemia, hypertension, congestive heart failure (CHF) and atrial fibrillation, and deep vein thrombosis (DVT) or pulmonary embolism (PE) [12].

The incidence of stroke has been reported in 5.7% of patients with severe COVID-19 and in 0.8% of patients with nonsevere infection [1]. The frequency of stroke detected in hospitalized COVID-19 patients was 1.1% associated with older age and stroke risk factors. Frequent cryptogenic strokes and elevated D-dimer support increased risk of thromboembolism in COVID-19 which is associated with high mortality [13].

The significant increase in D-dimer levels in patients with acute ischemic stroke suggests that COVID-19 can induce an inflammatory response and trigger a hypercoagulable state causing an acute ischemic stroke [14]. The hypercoagulable state in patients with COVID-19 supports the formation of small and/or large blood clots in many organs such as the brain, which have the potential to cause cerebrovascular disease (ischemic stroke or intracranial hemorrhage) [15].

D-dimer is a degradation product of fibrin cross linking. Increased D-dimer levels indicate global activation of hemostasis and fibrinolysis, and it is in line with the COVID-19-associated hypercoagulability hypothesis [16]. Increased D-dimer levels confirm the theories of endothelial activation and hypercoagulability, but other mechanisms are under investigation [10]. Some other plausible proposed mechanisms involved in stroke in COVID-19 are viral neurotropism, endothelial dysfunction, coagulopathy, and inflammation [11].

The possible effect of hypercoagulability as a cause of stroke after COVID-19 infection is still unclear although some recent case reports in young patients with few/no comorbid risk factors have the potential to lead to this hypercoagulability mechanism [17]. A COVID-19-associated hypercoagulable state from a proinflammatory process can be hypothesized as a possible contributing mechanism for the higher prevalence of embolic cryptogenic stroke presentation [5].

As COVID-19 infection has been found to lead to a hypercoagulable state, in this case, the role of anticoagulation is important. The pharmacological therapy provided was rivaroxaban oral anticoagulant.

## 4. Conclusions

In conclusion, this study reports a case of ischemic stroke after acute COVID-19 in a relatively young patient without preexisting cerebrovascular risk factors. Physicians should be aware, perform early identification, and treat stroke possibility on COVID-19.

## Data Availability

No data were used to support this study.
